# Interventions supporting cancer patients in making decisions regarding participation in clinical trials - a systematic review

**DOI:** 10.1186/s12885-022-10066-9

**Published:** 2022-10-26

**Authors:** Line Hillersdal, Zandra Engelbak Nielsen, Ane Taudorf Nørmark, Ann Knoop, Karin Piil

**Affiliations:** 1 Centre for Medical Science and Technology Studies, Department of Public Health, University of Copenhagen, Øster Farimagsgade 5, 1353 Copenhagen K, Denmark; 2 Department of Nursing and Nutrition, Faculty of Health, Copenhagen University College, Tagensvej 86, 2200 Copenhagen N, Denmark; 3 Department of Oncology, Centre for Cancer and Organ Diseases, Copenhagen University Hospital, Rigshospitalet, Blegdamsvej 9, 2100 Copenhagen, Denmark; 4 Department of Public Health, Aarhus University, Bartholins Allé 2, 8000 Aarhus C, Denmark

**Keywords:** Cancer, Decision support, Narrative synthesis, Oncology, Systematic review, Trial participation

## Abstract

**Objectives:**

Existing research on the perspectives of patients with cancer and health care professionals indicates that patient decision making on cancer clinical trial participation is a complex process and may be poorly understood, possibly compromising their decision to participate. This systematic review investigates interventions that support patients in their decision-making processes regarding whether to participate or not and assesses the qualities of the interventions, measures used and related outcomes.

**Methods:**

Six databases were systematically searched and only studies evaluating interventions that support the decision making of adult patients offered to enter a cancer clinical trial were included. Ten articles met the criteria and were analysed using a narrative synthesis approach.

**Results:**

The research focus of the included studies reflected the multifactorial nature of what constitutes support for patient decision making in terms of entering a cancer clinical trial. However, most interventions were based on the hypothesis that more information leads to support in decision making, and did not take other factors, such as the relationship to the clinical staff or relatives, the patients’ strong hope for therapeutic benefit or other existential needs into account. The interventions were primarily based on a specific tool, executed once, which seems to imply that decisions need only to be supported once and not at several time points throughout the decision process, and did not assess the importance of a patient’s family- or social relations. Moreover, few interventions focused on the patients’ counselling experience or assessed patient preferences in relation to decision making.

**Conclusions:**

The findings demonstrate a lack of research on interventions to support patients’ decision making that takes other factors, apart from improving knowledge of trials, into account. Limited evidence exists on the effectiveness of decision support interventions to improve the experience of support in adult patients with cancer. Interventions that take patient preferences in relation to decision making and the social context of decision processes into account need to be developed and assessed.

**Supplementary Information:**

The online version contains supplementary material available at 10.1186/s12885-022-10066-9.

## Background

Continued advancements in medical research have revolutionised treatments for cancer, and the number of clinical trials evaluating the effectiveness of new drugs continues to increase worldwide. Oncology clinical trials are central to establishing evidence that future patients can use to make informed treatment decisions. In mid-2021 there were 23,853 active clinical cancer trials registered in the U.S. National Library of Medicine’s ClinicalTrial.gov [1]. The number of participants involved in clinical cancer trials worldwide is unknown. In Denmark alone, a nation with about 5.8 million inhabitants, an estimated 34% of the 21,210 people participating in trials in 2019 were involved in cancer-related trials [2]. The inclusion of research subjects in clinical trials depends on multiple factors, such as the eligibility and willingness of patients to participate in clinical research but also the timing of trial protocols and the disease status of patients. It can be challenging for patients living with a cancer diagnosis to decide to whether or not to participate in a clinical trial. Moreover, the complexity of clinical trial protocols and procedures can be high and put extra demands on patients and/or their relatives.

Clinical pharmaceutical trials on humans are divided into four phases. Phase I trials assess drugs that have proven to be potentially effective in animal tests [3] and evaluate the safety and toxicity of the drugs at different dose levels. Early cancer trials most often include patients with cancer who have exhausted their treatment options but strongly wish to receive treatment [4]. Phase II trials are designed to evaluate the effectiveness of the drug in people with the disease or condition under study and to determine potentially adverse effects and risks associated with the drug. Phase III trials confirm and expand on safety and effectiveness results, compare the drug to standard therapies for the condition under study and evaluate its overall risks and benefits. Finally, phase IV trials evaluate the efficacy and safety of an already approved drug [3]. In research phases I and II of drug development, the therapeutic effect of the drug is unknown [4]. Due to the uncertainty of the treatment patients given the opportunity to participate in a trial need reliable information and substantial support to make informed treatment decisions. Making the choice to participate in a cancer clinical trial is known as a preference-sensitive decision [5]. Preference-sensitive decision is defined as a situation where the evidence for the superiority of one treatment over another is either not available or does not allow differentiation; in this situation, there are two or more valid approaches, and the best choice depends on how individuals value the risks and benefits of treatments [5]. This implies that the patient is required to choose a treatment despite the uncertainty of the outcome with respect to expected treatment effect or randomisation. In this context decision support may be beneficial for preference-sensitive treatment and screening decisions.

Informed consent is a cornerstone of ethical health care research and a requirement for conducting clinical trials [6]. The Harmonised Tripartite Guideline for Good Clinical Practice (ICH-GCP), which is an international ethical and scientific quality standard, helps protects the rights, safety and wellbeing of human subjects in clinical pharmaceutical trials and its principles have been incorporated in local legislation in the US, Canada, Japan, Europe and Australia [7]. In particular, the ICH-GCP emphasizes that patients must be fully informed, have the capacity and competence to make decisions, and voluntarily confirm their willingness to participate.

Additional knowledge about what makes patients decide to participate in a cancer clinical trial is crucial. Some studies report that patients have: a lack of knowledge on the rationale of the trial; a lack of understanding of the methodological processes of clinical trials, such as randomisation of treatment allocation; and that patients have fears about treatment efficacy, misunderstand the concept of equipoise and dislike discussing treatment uncertainty with health care professionals [8]. A recent published review by Nielsen and Berthelsen identified various factors that influenced the decisions patients with cancer make regarding participation in trials, and that some of these factors could potentially compromise the informed consent process, especially as to whether the patient has understood the trial information [8]. Patients reported that the positive or negative attitudes of their relatives towards participation influenced their decision to participate [9–12]. Furthermore, the patients’ trust in their physician’s recommendations and guidance was crucial and they rarely deviated from what their physician told them when deciding [12, 13]. Oncology and haematology nurses and physicians who reported similar findings in the Nordic countries stated that they experienced that their patients had an unwavering trust in their physician’s recommendations and that pressure from relatives on patients to participate in a trial caused ethical concerns [14, 15]. Other aspects that patients reported as influencing their decision were a strong hope that there would be a therapeutic benefit [10, 12–14, 16] and that they believed no other options than trial participation were available [9, 10, 13, 16–18]. Some of the studies found that the patients’ hope was based on a therapeutic misconception [19]. In cases where patients had misunderstood the purpose of the trial [17] or thought they had no other options, decisions were made quickly, the patient downplaying the trial information, risks and benefits [13, 16, 17]. In line with this, the health care professionals mentioned how patients with cancer often were motivated by unrealistic hope, despite the trial information they had received [14, 15]. Moreover, empirical research on informed consent for cancer clinical trial participation has shown that participants have higher expectations toward benefitting from experimental treatment than are usually warranted [20–23]. Furthermore, the timing of diagnosis influenced patients' decisions, meaning that newly diagnosed patients were emotionally overwhelmed, leading to greater difficulty in understanding crucial trial information [9]. In contrast, patients who have lived with the disease for more than a year found the decision to participate in trials to be more difficult than patients more recently diagnosed [12].

Existing research showing the perspectives of patients with cancer and of health care professionals indicates that the decision making of patients with cancer on trial participation is a complex process and that the patients’ reasoning may be poorly understood. These factors may compromise the decision making of patients to participate in research. This systematic review aims to review interventions carried out to support patients with cancer in their decision-making processes regarding whether or not to participate in in clinical cancer trials. To our knowledge, previously systematic reviews have been focusing on interventions targeted patients with varying diagnoses [24, 25] and not solely patients with cancer. Therefore, this review will contribute with knowledge to a field not explored in reviews before.

## Methods

This is a systematic review that employed the web-based software platform Covidence to screen and extract data and to manage the review [26], which also used the Mixed Methods Appraisal Tool (MMAT) for quality assessment, in accordance with Popay et al. [27, 28].

To identify how the decision-making process is conceptualised across the studies, we conducted a narrative synthesis based on Popay et al.’s guidelines [27]. The guidelines provide a framework of four stages: 1) developing a theory, 2) developing a preliminary synthesis, 3) exploring relationships within and between studies and 4) assessing the robustness of the synthesis. Each stage comprises various tools and techniques that are applicable based on the design of the included studies and the nature of the review (Supplementary Fig. S1).

### Stage 1: developing a theory

Following the narrative synthesis methodology introduced above we formulated a broad hypothesis to accommodate a complex understanding of the decision-making process based on the literature on factors influencing the decisions patients make to participate in a clinical cancer trial. A complex understanding of decision making entails understanding decisions as social processes influenced by the values and preferences of the individual patient, their significant others, the clinical encounter, and the broader social context. Recognising the influence of contextual and social factors, we considered it important to include interventions directed at individual patients as well as interventions directed at health professionals and family and community contexts. We also found the processual nature of making decisions significant and included support measures that addressed any decision support to strengthen our hypothesis that the intervention should meet individual characteristics, preferences, and circumstances. We wanted to investigate the various measures applied across the studies, including how and when they were applied in the study period. The inclusion of studies using various interventions across the inclusion process is reflected in our broad search strategy, which was designed to accommodate a comprehensive understanding.

### Search methods

Because of the scarcity of studies exploring interventions that support the decision making of patients with cancer when deciding to enter a cancer clinical trial, we conducted a systematic review employing a broad search strategy to uncover the existing evidence in the field. The review protocol has been registered in PROSPERO (ID CRD42020156577).

Based on population, intervention, comparison and outcome [29], the initial search strategy was developed and targeted specific databases in collaboration with a research information specialist (AL). The systematic searches, performed in CINAHL, PsycINFO, PubMed, Embase, Scopus, Social Science Citation Index – Web of Science and Sociological Abstracts databases on 7–11 November 2019, included a combination of free-text and subject heading searches related to overall search terms: neoplasm, cancer, oncology, haematology, patients with cancer (population), clinical trial, research subject, interventions, recruitment, research participation, trial enrolment, trial participation (intervention), decision, decision making, participation, non-participation, informed choice, decision support, decision aid, decision behaviour, and support techniques (outcome). No search terms covering comparisons were included due to the aim of the review, which was to explore a diverse range of interventions. The searches were limited to the following languages Danish, English, Norwegian and Swedish and publication date (2009–2019) but unlimited in terms of study design and included qualitative and quantitative studies (Supplementary Table S1). The 10-year period was chosen to strengthen the applicability of the review to clinical practice.

### Eligibility criteria

All study designs were eligible for inclusion if they were compatible with the review objective and met the inclusion criteria, outlined as follows: Full-text, peer-reviewed empirical studies with an adult (≥18 years) patient population. Study participants diagnosed with cancer were included if they were eligible to enter a clinical trial and the intervention aimed at supporting the decision making of the patient. We included all interventions supporting the trial inclusion process (e.g. information about the trial, giving consent and counselling) and those delivering the intervention (health care professionals, relatives and communities). Studies performed on a mixed cohort of patients with cancer and other patient populations were eligible, but only if the results directly related to the decision support of the patients with cancer could be extracted. Studies in patients with cancer without available clinical trials were excluded as were studies of decision support measures, without further intervention.

### Study selection

Two authors independently reviewed titles and abstracts. Four authors read the full text of potentially eligible studies and assessed them against the inclusion criteria. Disagreements were solved through discussion (see author contribution for further details).

### Quality assessment

The methodological quality of the included studies was assessed using MMAT [28, 30, 31], which is based on two general screening questions and five specific questions for each study design to concomitantly appraise qualitative, quantitative and mixed methods studies [31]. The questions were scored using “yes”, “no” or “can’t tell”, while questions not applicable to the specific study design were noted as N/A. All authors carried out the quality assessment individually. Potential disagreements were discussed to obtain consensus. The assessments were performed to gain insight into the methodological quality of the eligible articles and resulted in no further exclusions. To enhance evaluation of the methodological criteria, an overview was drawn up of the measurement tools applied in the included studies, allowing us to incorporate the underlying justification of the instruments (validity and standards) into the overall methodological considerations.

### Stage 2: developing a preliminary synthesis

At this stage we organised and described the findings to identify any crosscutting patterns. Textual descriptions of the studies and a tabulation of data extraction that grouped and described study characteristics and results were used to develop the framework for the analysis.

### Stage 3: exploring relationships within and between studies

The purpose of this stage is to explore differences within and across the studies in terms of factors that might explain variations, e.g. differences in effect across studies. Since exploring heterogeneity at this stage is also crucial we analysed the variables and subgroups in our findings to show the components for each study, as well as the overlap and differences between those components [27].

### Stage 4: assessing the robustness of the synthesis

This stage involves assessing the strength of the results of the synthesis to draw conclusions and generalise them in terms of the effect on different populations and/or contexts. We looked at the use of validity assessment in the studies using the quality assessment of each study that we had previously conducted earlier in the synthesis process. We also reflected critically on the synthesis process, discussing issues that had arisen along the way, which included doing a comparison of the theory we had initially developed with our preliminary results [27]. In the Discussion section the robustness of the synthesis is examined.

## Results

This section presents extracted data relevant to our study aim.

### Search results

**Fig. 1:**
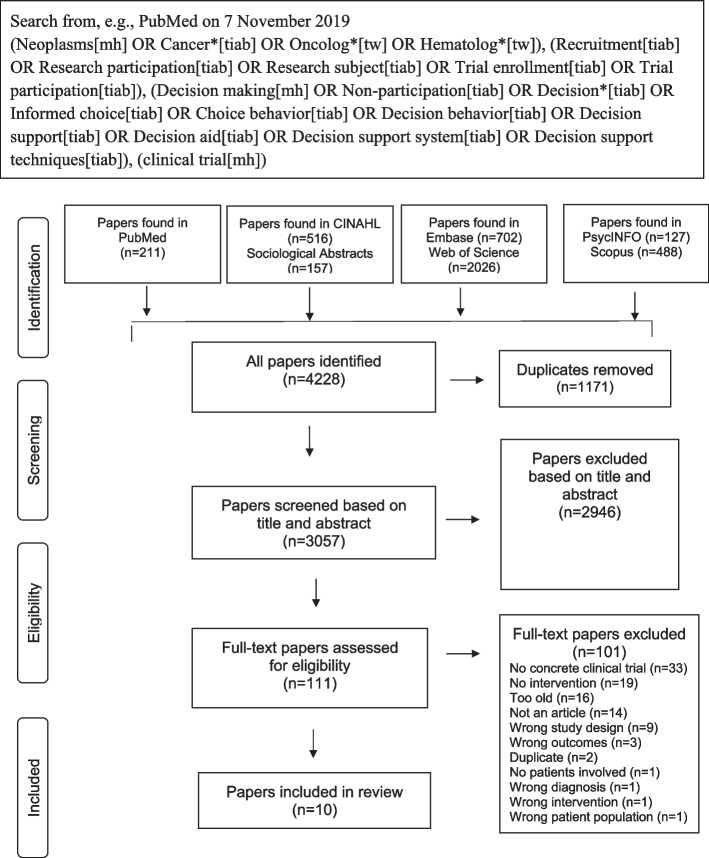
PRISMA flow chart

We identified 4228 studies in CINAHL, PsycINFO, PubMed, Embase, Scopus, Social Science Citation Index – Web of Science and Sociological Abstracts databases. After processing the retrieved items in Covidence [26], 1171 duplicates were removed, and we developed a preferred reporting items for systematic reviews and meta-analyses, or PRISMA, flow chart illustrating the screening process (Fig. 1). The 3057 studies that were included were screened by title and abstract. Of these, 2946 were excluded because they did not meet the inclusion criteria. The remaining 111 studies were assessed for eligibility based on a full-text review, which led to the exclusion of another 101 studies, leaving ten studies for further assessment.

### Preliminary narrative synthesis

This section presents an individual textual description of the ten studies and tables summarising study characteristics (Supplementary Table S2), the quality assessment (Table 1), the comparisons across studies (Table 2) and the measures applied (Table 3) in the studies.

**Table 1:** Quality Assesment

Category of study designs		Kass et al. 2009 [32]	Brown et al. 2012 [33]	Dear et al. 2012 [34]	Hoffner et al. 2012 [35]	Mills et al. 2014 [36]	Skovlund et al. 2017 [37]	Sundaresan et al. 2017 [38]	Tattersal et al. 2017 [39]	Kamen et al. 2018 [40]	Polite et al. 2019 [41]
Screening questions (for all types)	S1. Are there clear research questions?	+	+	+	+	+	+	+	+	+	+
	S2. Do the collected data allow the addressing of the research questions?	+	+	+	+	+	+	+	+	+	+
1. Qualitative	1. Qualitative										
	1.1. Is the qualitative approach appropriate to answer the research question?	N/A	N/A	N/A	N/A	+	+	N/A	N/A	N/A	N/A
	1.2. Are the qualitative data collection methods adequate to address the research question?	N/A	N/A	N/A	N/A	+	+	N/A	N/A	N/A	N/A
	1.3. Are the findings adequately derived from the data?	N/A	N/A	N/A	N/A	+	C/T	N/A	N/A	N/A	N/A
	1.4. Is the interpretation of results sufficiently substantiated by data?	N/A	N/A	N/A	N/A	+	C/T	N/A	N/A	N/A	N/A
	1.5. Is there coherence between qualitative data sources, collection, analysis, and interpretation?	N/A	N/A	N/A	N/A	+	C/T	N/A	N/A	N/A	N/A
2. Quantitative randomized controlled trials	2. Quantitative randomized controlled trials										
	2.1. Is random assignment appropriately performed?	-	N/A	+	+	N/A	N/A	+	+	+	N/A
	2.2. Are the groups comparable at baseline?	+	N/A	+	+	N/A	N/A	+	+	+	N/A
	2.3. Are there complete outcome data?	C/T	N/A	+	+	N/A	N/A	+	+	+	N/A
	2.4. Are outcome assessors blinded to the intervention provided?	-	N/A	+	C/T	N/A	N/A	+	+	+	N/A
	2.5. Did the participants adhere to the assigned intervention?	C/T	N/A	-	+	N/A	N/A	+	-	+	N/A
3. Quantitative nonrandomized	3. Quantitative nonrandomized										
	3.1. Are the participants representative of the target population?	N/A	N/A	N/A	N/A	N/A	N/A	N/A	N/A	N/A	C/T
	3.2. Are measurements appropriate regarding both the outcome and intervention (or exposure)?	N/A	N/A	N/A	N/A	N/A	N/A	N/A	N/A	N/A	+
	3.3. Are there complete outcome data?	N/A	N/A	N/A	N/A	N/A	N/A	N/A	N/A	N/A	+
	3.4. Are the confounders accounted for in the design and analysis?	N/A	N/A	N/A	N/A	N/A	N/A	N/A	N/A	N/A	-
	3.5. During the study period, is the intervention administered (or exposure occurred) as intended?	N/A	N/A	N/A	N/A	N/A	N/A	N/A	N/A	N/A	+
4. Quantitative descriptive	4. Quantitative descriptive										
	4.1. Is the sampling strategy relevant to address the research question?	N/A	+	N/A	N/A	N/A	C/T	N/A	N/A	N/A	N/A
	4.3. Are the measurements appropriate?	N/A	+	N/A	N/A	N/A	+	N/A	N/A	N/A	N/A
	4.4. Is the risk of nonresponse bias low?	N/A	+	N/A	N/A	N/A	+	N/A	N/A	N/A	N/A
	4.5. Is the statistical analysis appropriate to answer the research question?	N/A	+	N/A	N/A	N/A	-	N/A	N/A	N/A	N/A
**Score**		**3 (7)**	**7 (7)**	**6 (7)**	**6 (7)**	**7 (7)**	**2 (5) 5 (7)**	**7 (7)**	**6 (7)**	**7 (7)**	**5 (7)**

Note: Questions not applicable for the specific study design are marked NA; –, no; +, yes; C/T; can’t tell

**Table 2:** Comparison Across Studies

**Main theme**	**Subtheme**	Kass et al. 2009 [32]	Brown et al. 2012 [33]	Dear et al. 2012 [34]	Hoffner et al. 2012 [35]	Mills et al. 2014 [36]	Skovlund et al. 2017 [37]	Sundaresan et al. 2017 [38]	Tattersall et al. 2017 [39]	Kamen et al. 2018 [40]	Polite et al. 2019 [41]
Target population	Health personnel					X^¶^	X^¶^		X		
	Patients	X	X	X	X	X	X	X	X	X	X
	Relatives				X^§^						
Type of intervention	Website			X							
	Booklet							X		X	
	Question prompt list		X	X					X		
	Video	X			X					X	X
	Extra support from or training of research nurses/extra counselling					X	X				
Study design	Randomised controlled study	X		X	X			X	X	X	
	Quantitative descriptive study		X				X				X
	Qualitative study design					X	X				
Aim	Decision support	X	X	X	X	X	X	X	X	X	X
	Patient and health personnel’s satisfaction with the clinical trial discussion								X		
	Improve trial knowledge	X		X	X		X	X	X		X
	Increase recruitment	X		X		X	X			X	X
	Improve positive attitudes toward trials							X		X	X
Effect of intervention	Decision support			No^†^	Yes	No^†^	Yes	Yes	No	No	Yes
	Satisfaction with the clinical trial discussed								No		
	Improve trial knowledge	No^†^		NE^‡^	No		Yes	Yes	No		Yes
	Increase recruitment	No		No		NE^µ^	No			Yes	No
	Improve attitude toward trials							No		Yes	Yes
Time points for intervention	Trial discussed before consultation	X		X	X			X		X	X
	Trial discussed during consultation		X			X			X		
	Trial discussed after receiving information about trial						X				
Type of clinical trial	Phase I	X	X		X						ND^±^
	Phase II	X	X		X					X	ND^±^
	Phase III		X	X	X	X	X	X	X	X	ND^±^
Sex	Male	ND^±^	X	X	X	X	X	X	X	X	X
	Female	ND^±^	X	X	X		X		X	X	X
Ethnicity	Mainly Caucasian	X	ND^±^	ND^±^	X	X	ND^±^	ND^±^	ND^±^	X	X
Cancer diagnosis	Breast	ND^±^	X	X			ND^±^		ND^±^	X	
	Lung	ND^±^	X	X	X		ND^±^		ND^±^	X	X
	Gastrointestinal cancer	ND^±^		X	X		ND^±^		ND^±^	X	X
	Genitourinary	ND^±^	X		X		ND^±^		ND^±^		
	Prostate					X	ND^±^	X	ND^±^	X	
	Pancreas						ND^±^		ND^±^		X
	Sarcoma				X		ND^±^		ND^±^		
Country	Australia/New Zealand		X	X				X	X		
	Denmark						X				
	United Kingdom					X					
	USA				X					X	X
No earlier trial participation										X	

^†^ Weak evidence / insignificant; ‡p-value; NE=Not evaluated); ^§^ No evaluation on the effect on relatives, only the patients’ perception on the effect on their relatives; ^¶^Intervention targeted health personnel but only as a means for the intervention; ^µ^ Unable to draw conclusion as there was not control group in the study; ^±^Not described

**Table 3:** Applied Measures

Measures and target measurements	Cassileth Information Styles Questionnaire. Brown (2012) [33]	Self-developed Likert Scale Brown (2012) [33]	Control Preference Scale. Brown (2012) [33], Tattersall (2017/modified) [39] Polite 2019/ modified) [41]	Patient Satisfaction with the Consultation Scale. Brown(2012) [33]	Clinical Trials Attitude Scale^†^ Kamen (2018) [40]	Preparation for Decision Making Kamen (2018) [40]	Decisional Conflict Scale Dear (2012) [34] Sundaresan (2017) [38]	Quality of Informed Consent Scale. Hoffner (2012 [35]/ modified); Tattersall (2017) [39]^‡^ Sundaresan (2017/modified) [38]	Self-developed questionnaire^§^ Tattersall (2017) [39]
Validated measure	x		x	x		x	x	x	
Preference: details (about illness)		x							
Preference: knowledge (of trial)	x						x		
Preference: involvement in decisions			x	x			x		x
Preparedness for decision making(Usefulness of materials)						x			
Satisfaction with decision making									
Attitude to trials					x		x		x
Satisfaction with provider communication									
Willingness to participate							x		
Knowledge of trials (purpose of research, expectation of benefits and risks)							x	x	
Self-efficacy									
Symptoms of anxiety/depression							**x**		**x**

Measures and target measurements	Self-developed survey^¶^ Kass (2009) [32]	Self-developed questionnaire Hoffner (2012) [35]	Clinical trial questionnaire^μ^ Polite (2019) [41]	Self-developed Likert scales Polite (2019) [41]	Self-developed questionnaire Skovlund (2017) [37]	Self-developed feedback questions Mills (2014) [36]	Decisional Regret Scale±Sundaresan (2017) [38]	Satisfaction with Decision^‽^ Sundaresan (2017) [38]
Validated measure							x	x
Preference: details (about illness)								
Preference: knowledge (of trial)								
Preference: involvement in decisions								
Preparedness for decision making(Usefulness of materials)		x			x	x		
Satisfaction with decision making		x			x		x	x
Attitude to trials				x		x		
Satisfaction with provider communication		x			x			
Willingness to participate	x			x				
Knowledge of trials (purpose of research, expectation of benefits and risks)	x			x	x			
Self-efficacy				x				
Symptoms of anxiety/depression								

^†^ The actual measure could not be retrieved, as the source quoted was a conference paper: WellsKJ, JacobsenPB, QuinnGP, IsleyA, CormanM, SimpsonT. Development and validation of measures of patient perceptions regarding cancer clinical trials. Paper presented at: The American Public Health Association 138th Annual Meeting; 6–10 November, 2010; Denver, Colorado

^‡^ Elements from: Joffe S, Cook EF, Cleary PD, et al. Quality of informed consent: a new measure of understanding among research subjects. J Natl Cancer Inst. 2001;93:139–47

^§^ Elements from: Spielberger State-Trait Anxiety Inventory: ‘Satisfaction with Decision’, ‘Attitudes to RCT Trials Questionnaire. See. Spielberger CD. Manual for the state trait anxiety inventory (form Y). Palo Alto, CA: Consulting Psychologists Press, 1983

^¶^ Elements from Rodenhuis et al. A qualitative evaluation of patient motives to participate in a phase 1 trial to improve an informed consent procedure.1984

^µ ^ Measure drawn from Jacobsen PB, Wells KJ, Meade CD, et al. Effects of a brief multimedia psychoeducational intervention on the attitudes and interest of patients with cancer regarding clinical trial participation: a multicenter randomized controlled trial. J Clin Oncol. 2012;30: 2516-2521

^±^ Brehaut JC, O’Connor AM, Wood TJ, et al. Validation of a decision regret scale. Med Decis Making. 2003; 23:281–92

^‽^ Holmes-Rovner M, Kroll J, Schmitt N, et al. Patient satisfaction with health care decisions: the Satisfaction with Decision scale. Med Decis Making.1996;16:58–64

### Textual description of the included studies

In a 2009 study by Kass et al. [32], patients with cancer (*n* = 288) considering enrolment in an early phase trial were randomised between an intervention arm using a 20-min, computer-based multimedia presentation on early phase trials versus a control arm that received standard care; a pamphlet entitled ‘Taking Part in Clinical Trials: What Cancer Patients Need to Know.’ Patients in the intervention group watched the video in an empty room before meeting with the oncologist to receive more information about the trial. After the consultation, patients completed a survey in person or by phone on the purpose of the trial, expected benefits and risks and their possible decision about enrolment.

A 2012 study by Brown et al. [33] tested the utility of a clinical trial question prompt list (QPL) during oncology consultations that discussed phase I, II or III. The aim was to assess which questions patients were most interested in asking, whether they asked the questions and, if not, had the issue been raised by the physician. The QPL contained 33 questions, which patients saw before their consultation to assess which questions, they wanted to ask the oncologist. The consultation was recorded, and the patients filled out validated pre - and post-consultation questionnaires.

In a 2012 cluster randomised trial conducted by Dear et al. [34], 30 oncologists and their patients (*n* = 493) were randomly assigned to access the Australian Cancer Trials website, which provided general information about clinical trials and gave participants two QPLs or were assigned to standard care. The aim was to evaluate if the website increased the proportion of patients who brought up the subject of trial participation with the oncologist and if the website influenced the number and complexity of trial issues discussed, consultation length, enrolment, patient knowledge and the decisional conflict the patient experienced. Before the scheduled appointment with the physician, which was audio-recorded, the patients in the intervention group filled out a baseline questionnaire and were then automatically directed to the website. Two weeks later the patients were asked to complete an online follow-up questionnaire.

In a 2012 study conducted by Hoffner et al. [35], 45 patients were randomised to receive an intervention comprising a 20-min educational video explaining trials compared to 45 patients randomised to a control group. The aim was to assess the effect of the video on preparing patients with cancer to make decisions on participating in a clinical trial based on the patients’ understanding and perceptions, as well as the video’s impact on decision making and the patient-provider communication. The intervention group was given the educational video to take home, along with the clinical trial consent form, and asked to fill out a questionnaire after watching the video. The control group filled out the same questionnaire and was subsequently given the option of watching the video. In addition, patients in both arms were asked to fill out the questionnaire Perceptions of the Clinical Trials Video at their next visit.

In a 2014 multicentre study by Mills et al. [36], a trial were included to compare how patients expressed treatment preferences and how recruiters managed those preferences. In the trial research nurses were trained in exploring and managing patient treatment preferences when recruiting patients with prostate cancer to the ProtecT randomised controlled trial, which compared radical prostatectomy, radical conformal radiotherapy and active monitoring. The aim was to illustrate how recruiters facilitated the recruitment process. First, the patients were informed about their diagnosis, treatment, and the possibility to participate in a trial, in addition to being given an information sheet about ProtecT. One week later a research nurse specifically trained in recruitment conducted recruitment appointments with patients that were audio-recorded. Nine centres conducted 93 appointments that were subsequently analysed using a qualitative approach.

In a 2017 non-randomised, single-centre study, Skovlund et al. [37] examined whether a supplementary telephone interview conducted by research nurses could support the patients in making decisions about trial participation on a well-informed basis and, if so, whether the intervention was feasible. Of the 31 patients with cancer who were included as candidates for a complex clinical trial, 16 were told about the intervention at an appointment. Afterwards the physician informed the patient about a specific trial and a research nurse called the patients to conduct a structured, follow-up interview before the patient’s next appointment with a physician regarding consenting to the trial. The patient subsequently filled out a questionnaire about their experiences regarding the informed consent process, just as the health care professionals were interviewed in focus groups to evaluate the potential for future implementation of the intervention.

A 2017 study by Sundaresan et al. [38] aimed to determine the utility of a customised decision aid for men with prostate cancer considering participation in the RAVES trial, which compared radiotherapy with adjuvant versus early salvage. The multicentre study had 129 patients who were randomised to receive an information sheet about the RAVES with or without the decision aid, which was a booklet. After consenting to the study, the intervention patients filled out a baseline questionnaire and then read the information about the trial before reading the booklet. The control group did the same but were not given the booklet, which included specific information about the RAVES trial and general information about research participation. Patients subsequently had an appointment with a physician to make their final decision about participation. Patients were given questionnaires at the one- and six-month follow up visits.

Tattersall et al. [39] conducted a randomised, unblinded multicentre study in 2017 evaluating the effect of a self-developed QPL in patients (*n* = 88) considering enrolment in phase III cancer trials. At the start of their appointment with the physician or research nurse where trial participation was discussed, the study was introduced to the patients and patients consenting to the study were randomised. Of these, 45 were randomised to the intervention and received the QPL to review for 5 min before continuing to discuss the trial with the physician or research nurse. The list contained 51 questions about clinical trials that the patients might find useful to ask. Participants filled out questionnaires at baseline and within 3 weeks of deciding to participate in the trial.

In a 2018 study by Kamen et al. [40], 418 patients with various types of cancer eligible for a specific phase II or III trial were included and randomised 1:1 to receive a multimedia psychoeducational intervention on a DVD called “Clinical Trials: Are They Right for You?” designed to improve patient attitudes towards trials versus a booklet called “Taking Part in Cancer Treatment Studies” from the National Cancer Institute in the United States. The aim of the study was to compare the effect of the two interventions on decision support factors, attitudes toward trials and willingness to participate in a trial. The patients met with a study coordinator right before their appointment with the physician where the trial was discussed and were exposed to both abovementioned interventions while the study coordinator was present. The patients filled out questionnaires before baseline, post intervention and at the two-month follow up visit.

Polite et al. [41] conducted a quantitative pre- and post-test intervention study in 2019 to examine the effectiveness of an interactive health communication tool to evaluate, as a primary objective, whether the tool improved patient receptivity, willingness, knowledge and self-efficacy but also having a positive attitude towards trials. Patients with diverse types of cancer (*n* = 120) watched an interactive teaching video from the National Institutes of Health before trial participation was discussed. A study coordinator conducted a semi-structured debriefing interview at the end of the intervention session to understand the patient’s experience with the components of the intervention and the overall study experience. The patients also completed a pre- and post-intervention survey.

### Quality assessment of the studies

All 10 studies had a clear research question, and the collected data in each study seemed to be appropriate to address the respective research question (Table 1).

Only one study exclusively used a qualitative design [36] and succeeded in addressing all the quality points of interest. Polite et al.’s [41] study, which we classified as a predominantly quantitative nonrandomised study, did not account sufficiently for any confounders in the design and analysis. Moreover, it was not clear if the participants (16% of the eligible patients) were representative of the target population. A semi-structured interview was conducted to understand the patient experience, but how the interviews were analysed was not explained. In our assessment, based on a seven-point scale, the six quantitative RCTs scored six or seven points. Dear et al. [34] and Tattersall et al. [39] were awarded a score of six due to challenges involving participant adherence to the assigned intervention. In Dear et al.’s [34] study, 33 clinics declined to participate, while Tattersall et al.’s [39], participants decided to stop the trial prematurely due to low accrual rates. Kass et al.’s study showed that the groups of interest were comparable at baseline but failed to describe the other issues connected to the appraisal tool. Two studies were quantitatively descriptive, Brown et al. [33] meeting all the key points for this design, but it is unclear if Skovlund et al. [37] used a relevant sampling strategy due to the small sample size (*n* = 31) informing the research question. We are also in doubt as to whether Skovlund et al.’s statistical analysis was appropriate for addressing the research question as these considerations were lacking [37].

To sustain the overall methodological criteria evaluation, justification of the instruments is relevant. Overall, the research questions and the measurements the various studies applied vary and a wide range of questionnaires were used (Table 3). In half of the studies, they developed several of the measurement tools themselves with only a few studies using validated questionnaires [38, 40].

### Relationships and differences between the studies

This section contains two tables that group and compare findings across the ten studies, one summarising the findings in general (Table 2) and one presenting the specific measurement methods (Table 3).

### Moderator variables and subgroup analyses

A common feature of the ten studies was that none of them worked with a predefined concept of decision making or explored the concept. One study had an exclusively qualitative design, and the rest were quantitative and mainly RCTs. Almost all interventions were tool based with a predefined framework designed as a one-way and one-off tool made available to patients via, e.g. a website [34], a booklet [38, 40], QPLs [33, 34, 39] or videos [32, 35, 40, 41]. Only two interventions were dialogue-based [36, 37]. In the analysis of the interventions, it emerged that the main underlying assumption was that the decision support should be provided to address the patients’ lack of knowledge about clinical trials and the main purpose of the decision support was thus to improve the patients’ clinical trial knowledge and acquiring of knowledge was therefore seen as synonymous with exercising decision support [32, 34, 35, 37–39, 41].

The interventions were in all, but one study, targeted towards the individual patient alone. In one intervention, relatives were also given information but not systematically, and the intervention was only evaluated in terms of the patients and not their relatives [35]. Two studies included health care staff but only as a means of implementing the intervention [36, 37]. Furthermore, these two interventions evaluated the patient side only, except for one study, which conducted a group interview with the health care staff to assess the potential for future implementation [37].

In summary, the ten studies comprised 1592 patients who had various cancer diagnoses. Almost all of the studies were phase III trials [33–40], though four were also in phase II [32, 33, 35, 40], and three in phase I [32, 33, 35]. No studies focused solely on phase I or II, but several were only phase III [34, 36–39]. Nine studies listed the participants’ sex, seven of which contained a mixture of males and females [33–35, 37, 39–41]. Two studies focused solely on males [36, 38]. All ten studies were conducted in Europe and the US, and in the studies that listed participants’ ethnicity, they were mainly Caucasian [32, 35, 36, 40, 41], which may also be a result of the review’s inclusion and exclusion criteria. Only one study excluded patients if they had previously participated in a trial [40].

All ten studies focused on at least one of three related time points in the intervention: before the consultation that provided information about the trial [32, 34, 35, 38, 40, 41], during the consultation [33, 36, 39] or after the consultation [37].

All ten studies conducted interventions directed to support patients’ decisions [33–41], four of these found an effect [35, 37, 38, 41]. Seven of the studies aimed to improve patients’ trial knowledge through the intervention [32, 34, 35, 37–39, 41], and three found an effect of the intervention [37, 38, 41]. Of the six studies that aimed to increase recruitment to clinical trials through the intervention [32, 34, 36, 37, 40, 41], one had a positive effect [40]. All ten included studies investigated if the intervention could improve patient attitudes towards clinical trials, with one study showing an effect [41].

Studies with interventions that showed an effect on one or several parameters mainly used video [35, 40, 41], booklets [38, 40], QPLs [33, 34] and websites [34]. Extra counselling showed mixed results [36, 37]. No other patterns across the studies were identified in interventions that had shown an effect.

Table 3 lists the highly diverse variety of measures that the ten studies used to assess different aspects of the decision support given. Two measures were developed specifically for patients with cancer, the remainder were developed to assess patients’ general decision making. Moreover, many of the applied measures were self-developed questionnaires that drew on elements from previous studies or adopted modified aspects of a validated measure. Two of the studies applied a combination of measures [33, 38], and the rest applied one or two measures to assess the outcome of the intervention. The choice of measures indicates which outcomes the studies assessed. Most of the studies measured the importance of knowledge about trials for the recruitment or willingness to enter a trial. Other studies were interested in the clinical encounter and used a tool to assess the patients’ counselling experience or patient preferences in relation to decision making.

## Discussion

The ten intervention studies demonstrated a variety of decision support parameters applied during the decision process, and the research questions reflected the multifactorial nature of what constitutes support for patient decision making in terms of entering a cancer clinical trial. However, we found no definitions of decision support. In most cases, we did not find any references to existing cancer clinical trial decision-making literature, which suggests a multitude of factors that influence the outcomes [19, 42]. However, from on the descriptions of the interventions and the applied measures and outcomes, we identified the underlying assumptions about what might characterise decision support.

Based on our initial literature search and the hypothesis we developed, we worked from an understanding of decisions as social processes and that support measures should ideally reflect the complexity of trial decision making. We wanted to include studies that focused on both the patients’ individual decisions and the role of health care professionals, familial and community contexts in affecting patient decisions. Furthermore, we did not want to focus solely on one specific approach to decision support but included all interventions that supported decisions. In our search, we came across studies that worked with a similar conceptualisation of decision support, but in these cases the studies did not test an intervention and were thus excluded from the review. This review instead provides insight into how a variety of interventions promote support. We find that studies operate based on the understanding that more information leads to support in decision making and did not take other factors, such as the relationship to the clinical staff or relatives, the patients’ strong hope for therapeutic benefit or other existential needs into account [8]. The intervention focus of the ten studies is mainly on the individual patient and does not aim to assess the importance of a patient’s family- or social relations. And, finally, the interventions were based on a specific tool and executed only once, which seems to imply that decisions need only to be supported once and not at several time points throughout the decision process.

### Individual patient decision or decision as a social process

Given the complexity of deciding to enter into a clinical trial, counselling using particular conceptual frameworks for decision making, such as ‘Shared decision making’ or similar methodologies have been developed focusing on not only the patient, but also family and health care professionals who will jointly consider the scientific evidence and patient preferences and values before making a treatment choice [43]. The exchange is two-way, and the health care professional may not be the only primary source of information for patients [44]. A review by Biedrzycki [19] on factors influencing cancer clinical trial participation found that shared decision-making styles may also be more challenging. And similarly the one study that included relatives in the decision-making process also showed the lowest response [19]. In most of the ten studies examined, the inventions were mainly directed at the patient. Four studies also focused on the role of health care professionals or considered the role of relatives, but only one of them assessed these aspects as part of the intervention [39].

### Information as decision support

Our focus on including a variety of decision support measures also meant that we expected to find specific decision-making concepts, such as the shared decision-making model or self-made learning and counselling programmes as examples of what to assess in the interventions. However, in the included studies, predominately interventions that provided more information to the patient were assessed. The focus on the content and structure of information as decision support and on assessing the level of knowledge gained has been criticised for neglecting the social context of decision processes. A review done by Gillies and colleagues [45] on decision aids found that discussions, compared to information received during the informed consent process, increased understanding, which suggests that it is not just information but the chance to reflect and share thoughts along the way that aid the decision-making process. Among the included studies three used a QPL, which is one way to give patients the opportunity to discuss their thoughts on trial participation. One of the cases, however, reported that patients did not always ask the questions they had rated the most important to ask [33]. This might suggest that health care professionals need to further assist in facilitating QPLs to better address the patients’ needs. Overall, a greater focus on patient preferences seems important. Only a few of the studies assessed patient preferences. When faced with uncertainties, such as advanced cancer cases, more information is often not preferred [46], as patients participate due to trust, belief in a cure or the wish to be able to some kind of actions.

### Recruitment and the aim of providing decision support

Many clinical trials face recruitment issues, and across the studies included the aim to increase recruitment is often assessed as part of decision support. While finding ways to improve recruitment is interesting, we find that the availability of decision support is also successful if it leads potential subjects to taking an informed stance to *decline* to participate. The focus on recruitment as a signpost to measure good decision support obscures what characterises good decision support from the patient and peer perspectives. One study highlighted this aspect by pointing out that, though the intervention modified patients understanding of belief and purpose of early phase 1 trials it did not lead to the inclusion of more patients [41]. Exploring why some patients decline to participate in a trial could serve to further inform the development of decision aids.

### The decision process

In all cases the studies offered the intervention at only one time point, which is why they did not examine the decision-making process as potentially having more than one time point. Furthermore, in almost all cases, the patients were only given a short amount of time to reflect on the material they received. However, in one of the studies, patients were allowed to watch the video at home with family members. A review by Robertson and colleagues [47] on decision making in paediatric oncology underlines that the importance of the decision to enrol in a clinical trial should be considered a process rather than a singular event. They recommended spending more time with families when they were making the decision and that the informed consent process should be taken in more stages, with more time to deliberate [47].

### Study design in decision support research

Every study except one was a standard RCT and applied a structured design. Choice of methodology affects what can be tested and learned about decision support. We find this reflected in what could be termed an instrumental approach to decision support. In most cases patients were given a book, video, or list of questions to deliberate in a very short time span and then assessed immediately after, though sometimes at a later stage as well. This indicates that the aim was to avoid confounders and test only the specific tool administered and does not give focus to how decisions tend to be more social achievements in which patients consult both professionals and relatives over time. Accordingly, additional thought needs to be given to the preference of the patients and address which research design is best suited for assessing decision support.

## Methodological discussion

We systematically and thoroughly applied Popay but also selected aspects of the framework most suitable to our aim. In the process we used investigator triangulation in involving multiple researchers in collecting or analysing data to support and qualify our findings. A review is a retrospective analysis and in formulating our hypothesis we find ourselves situated in contemporary clinical experience and in recent research that has influenced our conceptualisation of decision support. Critiquing earlier studies for not reflecting our contemporary understanding was not our intention but the recent rise in decision-making literature demonstrated to us how little of that knowledge has been used to design interventions.

We assessed the scientific quality of the studies according to MMAT and found that the methodological quality was generally good. However, the different purposes, nature and design of the questionnaires used may raise the concern that the gold standard questionnaire for user-experience/involvement in clinical trials has yet to be defined relying on psychometric validation procedures. This also calls into question the relevance of using appraisal tools developed for quantitative methods when evaluating patients' needs and willingness to participate in a clinical trial. Another impediment when making recommendations is the lack of critical reflection in the papers on the questionnaires that were applied. Moreover, the interventions conducted were not differentiated according to phase I–III trials, which may otherwise have revealed differences in patient preferences and their need for support and clarification as to whether there is a need to tailor decision aids further. In addition, patients who had participated in a trial previously were not excluded from the trials, except in one study [37], which might be viewed as an important bias as previous trial experiences might have influenced the decision-making process and consequently the intervention effect.

## Study limitations

The inclusion in the review of various interventions and target groups supported our understanding of the complexity of the decision-making process and the underlying assumptions that go with various measures and outcomes. However, this broad view did not allow us to evaluate more specifically with respect to the detailed effect of specific measures or outcomes. Hence, we were challenged to make a firm conclusion due to the diversity of study design for the included studies.

In using the narrative synthesis framework by Popay, we selected aspects of the framework suitable to our aim. Still, due to time constraints, we left out the suggestion to contact the authors of the included studies for a review of our synthesis.

## Clinical implications

The findings of this review emphasise the need to address the conceptual understanding of decision support, with greater clarification ultimately benefiting the patients. The aim of future interventions will not primarily be directed at providing more information or improving accrual rates but will address values and preferences of patients, in addition to other factors involved in making treatment decisions. As a result, we recommend involving patients and the public in the development of future research design. This is highly warranted since research and treatment are becoming increasingly more entangled in daily clinical practices, especially within cancer treatment.

## Conclusion

None of the studies defined decision support. The interventions mainly focused on individual patients and measured the patients’ improvement of knowledge about trials related to recruitment or willingness to enter a trial. The findings show a lack of research on interventions to support patients considering trial participation that takes other factors into account, like their relationship to the clinical staff or relatives, patients’ strong hope for therapeutic benefit or other existential needs. Limited evidence exists on the effectiveness of decision support interventions to improve the experience of support in adult patients with cancer. Few interventions focused the patients’ counselling experience or assessed patient preferences in relation to decision making. More interventions that take the social context of decision processes into account must be assessed.

## Supplementary Information

**Additional file 1: Supplementary Fig. 1.** Synthesis process.

**Additional file 2: Supplementary Table S1.** Search strategy.

**Additional file 3: Supplementary Table S2.** Characteristics of the included studies.

## Data Availability

Data comprise research articles sourced from publicly available databases at the Danish Royal Library system (https://soeg.kb.dk/).
