# Supplementary figures and images for: Interventions supporting cancer patients in making decisions regarding participation in clinical trials - a systematic review
Source: BMC Cancer. 2022 Oct 26;22:1097. doi: 10.1186/s12885-022-10066-9 (PMC9609242; doi:10.1186/s12885-022-10066-9)

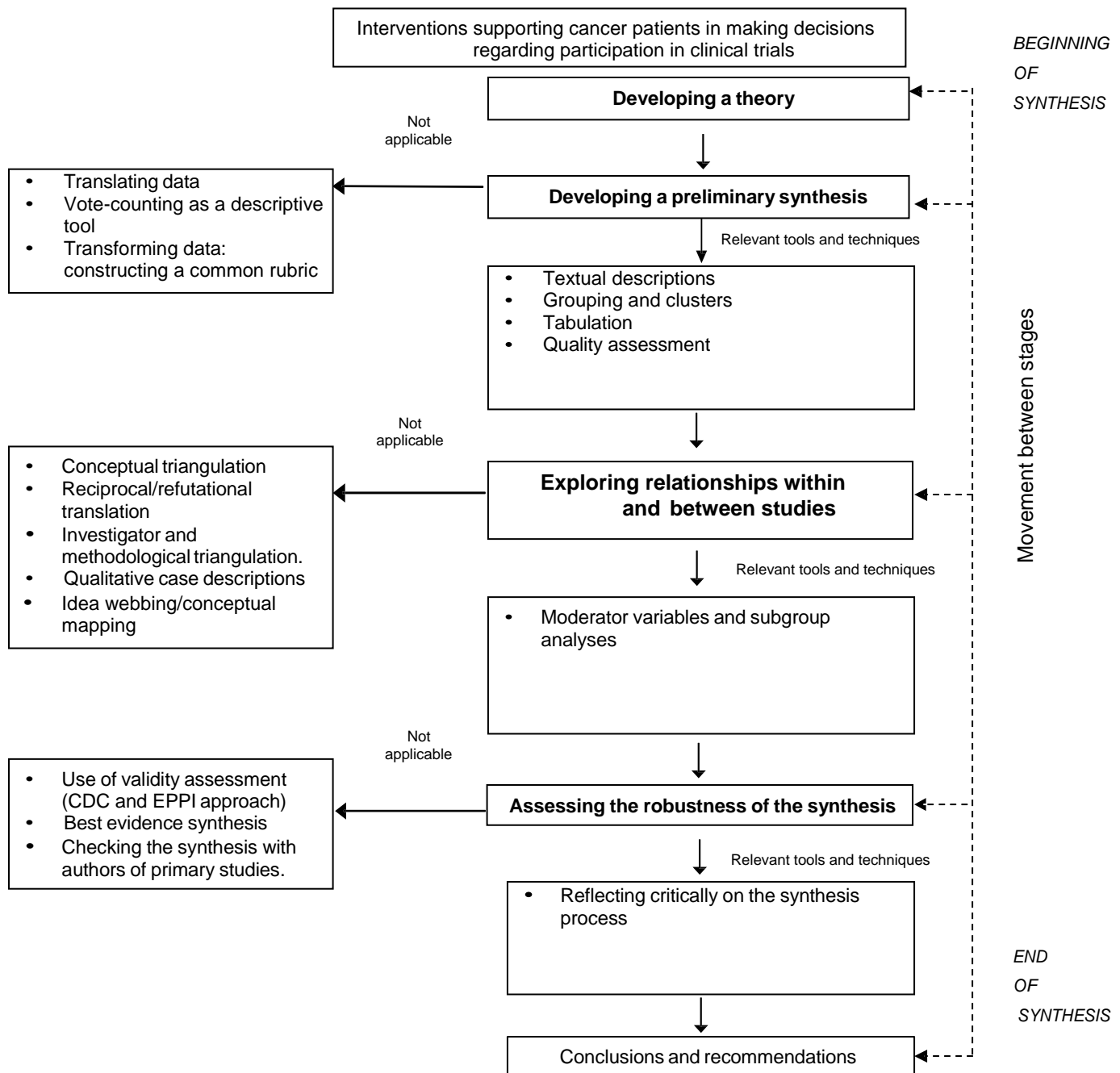

Supplementary Figure 1: Synthesis Process

Supplement: Supplementary file 1 — Additional file 1: Supplementary Fig. 1. Synthesis process. [file 12885_2022_10066_MOESM1_ESM.pdf]
