# Supplementary material for: Interventions supporting cancer patients in making decisions regarding participation in clinical trials - a systematic review
Source: BMC Cancer. 2022 Oct 26;22:1097. doi: 10.1186/s12885-022-10066-9 (PMC9609242; doi:10.1186/s12885-022-10066-9)
Supplement: Supplementary file 2 — Additional file 2: Supplementary Table S1. Search strategy. [file 12885_2022_10066_MOESM2_ESM.pdf]

Supplementary Table S1. Search Strategy

Database: PubMed

Search on the Nov 7th, 2019, imported to Zotero

**1**	(Neoplasms[mh] OR Cancer*[tiab] OR Oncolog*[tw] OR Hematolog*[tw])	**3.797.585**
**2**	(Recruitment[tiab] OR Research participation[tiab] OR Research subject[tiab] OR Trial enrollment[tiab] OR Trial participation[tiab])	**123.748**
**3**	(Decision making[mh] OR Non-participation[tiab] OR Decision*[tiab] OR Informed choice[tiab] OR Choice behavior[tiab] OR Decision behavior[tiab] OR Decision Support[tiab] OR Decision aid[tiab] OR Decision support system[tiab] OR Decision Support Techniques[tiab])	**488.608**
**4**	(clinical trial[mh])	**332.608**
**5**	1 AND 2 AND 3	**616**
**6**	5 AND 4	**211**

Database: Sociological Abstracts

Search on Nov 7th, 2019, imported to Zotero

**1**	(Neoplasms OR Cancer* OR Oncolog* OR Hematolog*)	**16.583**

**2**	(Recruitment OR "Research participation" OR "Research subject" OR enrollment OR participation OR Non-participation)	**156.647**
**3**	("Decision making" OR Decision* OR "Informed choice" OR "Choice behavior" OR "Decision behavior" OR "Decision Support" OR "Decision aid" OR "Decision support system" OR "Decision Support Techniques")	**161.786**
**4**	“Clinical trial”	**1364**
**5**	1 AND 2 AND 3	**3397**
**6**	5 AND 4	**157**

Database: Psychinfo

Search on the Nov 10h 2019, imported to Zotero

**1**	(Neoplasms OR Cancer* OR Oncolog* OR Hematolog*)	102,064
**2**	(Recruitment OR "Research participation" OR "Research subject" OR enrollment OR participation OR Non-participation)	139,551
**3**	("Decision making" OR Decision* OR "Informed choice" OR "Choice behavior" OR "Decision behavior" OR "Decision Support" OR "Decision aid" OR "Decision support system" OR "Decision Support Techniques")	251,979
**4**	“Clinical trial”	19,641
**5**	1 AND 2 AND 3	1,196

**6**	5 AND 4	**127**

Database: EMBASE

Search Nov. 10th 2019, imported to Zotero

**1**	(Neoplasms OR Cancer* OR Oncolog* OR Hematolog*)	**3.774.183**
**2**	(Recruitment OR Research participation OR Research subject OR Trial enrollment OR Trial participation)	**183.986**
**3**	(Decision making OR Non-participation OR Decision* OR Informed choice OR Choice behavior OR Decision behavior OR Decision Support OR Decision aid OR Decision support system OR Decision Support Techniques)	**687.006**
**4**	Clinical trial	**1.510.312**
**5**	1 AND 2 AND 3	**1390**
**6**	5 AND 4	**(702**

Database: Web of Science

Search Nov. 12th 2019, imported to Zotero

**1**	ALL= (Neoplasms OR Cancer* OR Oncolog* OR Hematolog*)	**3.654.342**
**2**	(Recruitment OR Research participation OR Research subject OR Trial enrollment OR Trial participation)	**1.223.226**

**3**	(Decision making OR Non-participation OR Decision* OR Informed choice OR Choice behavior OR Decision behavior OR Decision Support OR Decision aid OR Decision support system OR Decision Support Techniques)	**1.425.475**
**4**	Clinical trial	**848.361**
**5**	1 AND 2 AND 3	**8098**
**6**	5 AND 4	**2026**

Database: Scopus

Søgning Nov. 10th 2019, imported to Zotero

**1**	TITLE-ABS-KEY (*neoplasms* OR *cancer** OR *oncolog** OR *hematolog**)	**4.139.883**
**2**	TITLE-ABS-KEY (*recruitment* OR *"Research participation"* OR *"Research subject"* OR *"Trial enrollment"* OR *"Trial participation"*)	**202.672**
**3**	TITLE-ABS-KEY (*"Decision making"* OR *non-participation* OR *decision** OR *"Informed choice"* OR *"Choice behavior"* OR *"Decision behavior"* OR *"Decision Support"* OR *"Decision aid"* OR *"Decision support system"* OR *"Decision Support Techniques"*)	**1.576.579**
**4**	TITLE-ABS-KEY (*"Clinical trial"*)	**1.452.050**
**5**	1 AND 2 AND 3	**866**
**6**	5 AND 4	**488**

Database: CINAHL

Search November 12th 2019, imported to Zotero.

**1**	(Neoplasms OR Cancer* OR Oncolog* OR Hematolog*)	**753.431**
**2**	(Recruitment OR Research participation OR Research subject OR Trial enrollment OR Trial participation)	**64.392**
**3**	(Decision making OR Non-participation OR Decision* OR Informed choice OR Choice behavior OR Decision behavior OR Decision Support OR Decision aid OR Decision support system OR Decision Support Techniques)	**256.162**
**4**	Clinical trial	**252.692**
**5**	1 AND 2 AND 3	**1125**
**6**	5 AND 4	**516**
