# Supplementary material for: Interventions supporting cancer patients in making decisions regarding participation in clinical trials - a systematic review
Source: BMC Cancer. 2022 Oct 26;22:1097. doi: 10.1186/s12885-022-10066-9 (PMC9609242; doi:10.1186/s12885-022-10066-9)
Supplement: Supplementary file 3 — Additional file 3: Supplementary Table S2. Characteristics of the included studies. [file 12885_2022_10066_MOESM3_ESM.pdf]

Supplementary table S2. Characteristics of the included studies

Reference	Study objective	Intervention	Study participants	Results of interest
**Kass et al. (2009)**<br><br>An Intervention to Improve Cancer Patients' Understanding of Early-Phase Clinical Trials	To test a computer-based multimedia intervention to explain clinical trials and enrollment decisions against an informational pamphlet	Intervention group: Self-directed, narrated, 20-minute computer-based presentation on early phase trials, including a consultation roleplay with suggested questions to ask the physician: the patients viewed it in an empty room before the appointment with their physician for trial information<br><br>Control group: An informational pamphlet called Taking Part in Clinical Trials: What Cancer Patients Needs to Know developed by the National Cancer Institute was given to patients to review before their appointment with their physician for trial information<br><br>Post-consultation survey for both groups	Cancer patients from three sites in the US referred to an evaluation with an oncologist regarding possible participation in an early phase trial and if their oncologists were willing to have the consultations audiotaped<br><br>Intervention group: (n=70)<br>Control group: (n=60)<br><br>No description of participant characteristics, except that most were Caucasian	Majority from both groups mistakenly reported that the purpose was efficacy<br><br>More patients in the invention group stated correctly that the purpose was related to safety.<br><br>More patients from the intervention group believed they might benefit from the drug or believed their physician thought it would be a good idea and would benefit them<br><br>No significant difference in the belief that the trial would affect their or others' cancer disease or regarding expected side effects, but many believed they would experience a long-term benefit or cure and have no or minor side effect than believed this to be true for other cancer patients<br><br>Patients who declined trial participation were significantly more likely to believe that their disease would get worse or experience no change, and 50% of patients enrolled expected a long-term effect or cure, compared to patients who did not enroll (13%)<br><br>Other significant factors for enrollment were expectation for long-term benefit, older age, being offered a phase 1 rather than a phase II trial<br><br>No significant difference for enrollment decisions between the two groups

Reference	Study objective	Intervention	Study participants	Results of interest
**Brown et al. (2012)**<br><br>Testing the utility of a cancer clinical trial specific Question Prompt List (QPL-CT) during oncology consultations	To test the utility of the Question Prompt List to:<br><br>1) assess which questions patients were most interested in asking<br><br>2) find out whether or not these questions were asked<br><br>3) find out whether oncologists discussed information related to questions patients wanted to ask but did not	Patients used a QPL-CT during cancer audio-recorded consultations while discussing with their oncologist<br><br>QPL-CT contains 33 questions in 11 categories:<br>Understanding My Choices;<br>Finding Out More About the Trial;<br>Understanding the Trial's Purpose and Background;<br>Understanding the Possible Risks;<br>The Difference Between Going on the Trial and Standard Treatment;<br>Understanding How the Trial Is Being Carried Out;<br>Understanding Possible Conflicts of Interest; Understanding My Right to Join the Trial or Not;<br>and Alternative Therapies<br><br>Pre- and post-consultation questionnaires	Eligible patients for therapeutic phase I, II or III trials from three participating outpatient clinics in New York:<br>Breast cancer (n=10)<br>Lung cancer (n=10)<br>Genitourinary cancer (n=10)<br><br>Male (n=15), female (15), average age 63 years, mostly married (66%) and university educated (66%)	All QPL-CT questions were selected by at least one-third of participants<br><br>Participants mostly wanted and asked questions about personal trial benefit<br><br>25 patients filled out the QPL-CT: Phase 1 trial (n=7), phase II trial (n=12), phase III trial (n=6)<br><br>Patients (75%) wanted to ask these three questions the most:<br>1. Question 7: What is already known about the treatment success? (83%)<br>2. Question 13: What are the risks of taking the new treatment?' (80%)<br>3. Question 11: 'Has the benefit of the new treatment been proven in people like me?' (77%)<br><br>Most participants (79%) preferred participatory decision making<br><br>Participants asked questions about benefits and side effects, suggesting that QPL-CT may be a useful tool to aid patients in initiating conversations about realistic expectations
**Dear et al. (2012)**<br><br>Impact of a cancer clinical trials web site on discussions about trial participation: A cluster randomised trial	To evaluate if access to the Australian Cancer Trials (ACT) web site:<br><br>1) increased the proportion of patients who discussed the	Intervention group: Filled out an electronic baseline questionnaire and was then automatically directed to the ACT website for the intervention; this was done before the scheduled appointment with the physician.<br><br>The ACT website provides general information about clinical trials and	84 eligible clinics in New South Wales and Victoria, Australia<br><br>30 medical oncologists at 30 clinics participated and recruited 493 patients (50% with early cancer)	Proportion of patients with whom the possibility of participation in any clinical trial was discussed; the number of issues about trials discussed (18 coded), the overall complexity of the trial discussion and consultation length<br><br>Overall complexity of the trial discussion was coded as extended when the trial purpose and rationale were explained or as basic when only minimal information was given

Reference	Study objective	Intervention	Study participants	Results of interest
	possibility of joining a clinical trial with their oncologist<br><br>2) had an impact on the number and complexity of trial issues discussed, consultation length, accrual rates to recruiting trials, patient knowledge about randomised clinical trials and patients' decisional conflict whether to join a trial	two question prompt lists that suggest questions patients can ask their physician about clinical trial participation<br><br>Control group: Received the standard information provided by oncologists before their scheduled appointment<br><br>Audio recordings of oncologist–patient consultations<br><br>All patients were asked to complete an online follow-up questionnaire	61 medical oncologists at 33 clinics declined to participate	Secondary outcomes obtained from patient questionnaires were the proportion of patients who consented to a trial, had knowledge about randomised trials and a decisional conflict about the possibility of joining a clinical trial<br><br>Participation in a trial was discussed:<br>Intervention group: 46% (84/183)<br>Control Group: 34% (72/215)<br><br>2% in intervention group invited to join a clinical trial compared with 5% in control group<br><br>No significant difference in the proportion invited to join a trial and the proportion that consented to a trial, reflecting that there are many factors that influence a physician's decision to invite a patient to join a trial and the patient's decision to consent<br><br>Physicians' lack of awareness about recruiting for clinical trials and their difficulty in adequately informing patients about trials are also barriers<br><br>There was no significant difference in mean consultation length (p=0.69), the mean number of issues about clinical trials discussed (p=0.96), whether the patient raised the possibility of participation in a clinical trial (p=0.81) and the percentage of consultations that included an extended clinical trial discussion (p=0.23)<br><br>QuIC section A and B: No significant difference between CG vs IG; no measurable impact on patient knowledge about clinical trials

Reference	Study objective	Intervention	Study participants	Results of interest
**Hoffner et al. (2012)**<br><br>Entering a Clinical Trial: Is it Right for You?	To assess the effect of an educational video in preparing cancer patients for decisions regarding clinical trial participation<br><br>1) The video's effects on patients' understanding and perceptions<br><br>2) The video's impact on decision making, patient-provider communication and patient satisfaction with video	Educational, 20-minute video designed to explain clinical trials clearly, simply and in a balanced way<br><br>Intervention group: Were given the video and the clinical trial consent to take home together; questionnaire completed after viewing the video<br><br>Control group: Completed questionnaire before viewing video<br><br>After their next visit at the clinic where the trial was discussed with the oncologist and trial consent was planned<br><br>All patients asked to fill out a questionnaire	Cancer patients considering participation in a phase I, II or III treatment trial from three clinics (at same hospital in the US<br><br>Intervention group (n=45 included, n=38 completed)<br>Control group: (n=45 included n=30 completed)<br>Attrition rate (n=13, 14.4%)<br><br>Mean age: 55<br><br>Diagnosis:<br>Sarcoma 29.9%,<br>Colon cancer 19.5%<br>Lung cancer 3.0%<br>Other GI 11.7%<br>Genitourinary 3.9%<br>Other 11.7%<br>Missing 10.4%<br><br>Women 57%<br>Caucasian 93%<br>College educated 57%<br>In a relationship 82%<br><br>Previous clinical trial participation:<br>Unknown (n=1)<br>No (n=25)/ Yes (n=13)	Perceptions of the clinical trials video: 72 patients completed the questionnaire: 89% found watching it worthwhile; 85% an important source of information, 81% felt better prepared to communicate with health professionals about the trial; 26% found that it generated new questions to ask their physician or nurse; one-third felt the video influenced their decision regarding participation (twice as many men as women); 89% found it helped family and friends to understand clinical trials better<br><br>A higher percentage of men (40%) compared to women (19%) found that the video helped them in deciding whether or not to participate.<br><br>Approximately half of the sample watched the video with family and/or friends, of those, 89% found it helped family and friends to better understand clinical trials<br><br>One-quarter of participants identified new questions to ask their doctor or nurse after watching the video

Reference	Study objective	Intervention	Study participants	Results of interest
**Mills et al. (2014)**<br><br>Training recruiters to randomized trials to facilitate recruitment and informed consent by exploring patients' treatment preferences	To illustrate how strategies used by recruiters might be used to inform methods to optimise recruitment	Research nurses received training using documents, individual and group discussions, and roleplay for recruiter to elicit and explore treatment preferences.<br><br>Patients were informed about diagnosis, treatments, and the need for a RCT and were given a written patient information sheet<br><br>About a week later, a research nurse conducted a recruitment appointment (the intervention)<br><br>The research nurses used a checklist to ensure that essential information was covered during the conversation with the patient (conversations ranged from 21–120 minutes).<br><br>The appointment continued until the recruiter was satisfied that the patient was sufficiently prepared to consider all the treatments to permit randomisation or was felt to be informed enough to choose a particular treatment	Potential candidates for a trial comparing prostatectomy, radical conformal radiotherapy, and active monitoring in 1,500 men 50–69 years of age with localised prostate cancer<br><br>A sample of 93 recruitment appointments from all nine centres was included<br><br>Men 100%<br>Mean age 62 years<br>Age range 50–69 years<br>Caucasian 72%<br>In a relationship 82%	Treatment preferences were expressed in 74 of the 93 recruitment appointments<br><br>50% of the patients who originally stated a treatment preference ended up deciding to participate in the RCT<br><br>Recruiters used three key strategies:<br>1. Elicit and acknowledge the rationale for the preference<br>2. Balance participant views about treatment<br>3. Emphasise need for participant to consider all treatments and equipoise<br><br>It is possible to train trial recruiters to elicit and address patient treatment preferences to help them to consider participation in the trial; they do this more consistently than recruiters who do not receive this type of training<br><br>Without specific training and guidance, recruiters are likely to accept initial preferences without understanding the reasoning behind them
**Skovlund et al. (2017)**<br><br>Phone consultation during clinical cancer trials: The project nurse's	To investigate whether a supplementary telephone interview:	Intervention group: Research nurses conducted telephone consultations with protocol-specific insight (structured telephone interview) as a follow-up between	Randomised trials with standard vs. experimental treatment<br><br>Controlgroup: (n=15)	The questionnaire survey showed that the telephone intervention had limited significance on the decision to participate in the trial, but great significance in relation to feeling safe (ca 75%) and well-informed (ca 65%)

Reference	Study objective	Intervention	Study participants	Results of interest
follow-up on the information before consent to clinical trials - the patient's perspective<br><br>Original title: [Telefonkonsultation ved kliniske kræftforsøg: Projektsygeplejerskens opfølgning på informationen før samtykke til kliniske forsøg – patientens perspektiv]	1) gives the patient an experience of having qualified their basis of understanding prior to possible trial participation<br><br>2) can be a future supplement to ensure that patient participation in trials takes place on a well-informed basis<br><br>3) gives patients the experience of having a qualified understanding of a trial prior to making a decision<br><br>4) is feasible	the physician appointment where trial information was given, and the physician appointment where consent/decline was given<br><br>Control group: Standard procedures<br><br>All patients completed a questionnaire on how they experienced the informed consent process	Intervention group: (n=16)<br><br>Complex clinical trials: diary keeping, QoL and/or many additional examinations, unknown treatment or extensive screening programme<br><br>Men (n=16), women (n=12)<br><br>Mean age 65 years<br>Age range 28–69 years<br><br>In a relationship (20)<br>Single (8)<br><br>Former participation in a clinical trial 1%	A sizeable proportion of the patients indicated in the telephone conversation that they had already decided<br><br>Even though the patient reported being well-informed by the information the physician provided, the research nurses noted that only half the patients appeared to be well-informed at the beginning of the phone conversation<br><br>Has helped in relation to putting the clinical trial in relation to everyday life<br><br>It is unknown whether the higher degree of understanding is due to: the intervention alone, that the patients had read the patient information, had time to digest the information, had talked to relatives or the fact that they had now talked to professionals twice (both the doctor and the study coordinator)<br><br>The intervention had limited impact on decision making, but had a major impact on the feeling of being well-informed and confident; the patients lacked knowledge about specific trial procedures

**Sundaresan et al. (2017)**<br><br>A randomised controlled trial evaluating the utility of a patient Decision Aid to improve clinical trial (RAVES 08.03) related decision-making	Primary aim:<br><br>to determine the utility of a customised decision aid for men considering participation in a prostate cancer, radiotherapy related RCT to determine whether providing a decision aid provision reduces decisional conflict regarding participation in RAVES<br><br>Secondary aims:<br><br>to evaluate whether the decision aid increased knowledge regarding RCTs and RAVES, improved understanding of and attitudes towards RAVES, decreased anxiety, improved decisional satisfaction, reduced decisional regret and influenced recruitment to RAVES	Eligible patients for the RAVE trial were invited to the DA study<br><br>All study material was then given to the patient (DA study information and consent form, baseline questionnaire, RAVES information sheet, DA booklet, RAVE information sheet and blank notebook)<br><br>It was blinded to personnel if the material contained the intervention or non-intervention<br><br>Patients were not introduced to the RAVE trial before or at this time point; after consenting to the DA study, the patients filled out the baseline questionnaire and then read the standard information about the RAVE trial and the DA booklet versus no-intervention for the control group.<br>Test time points:<br>Questionnaires were administered at baseline, one month and six months<br>(follow-up questionnaires at one month and six months after baseline)<br><br>Decision aid booklet: customised for men with prostate cancer considering participation in the RAVE trial; included evidence-based information on the pros and cons of the two management options being compared in RAVES	129 men (prostate cancer and positive margins and/or pathological stage T3 disease at radical prostatectomy) were randomised to the IG with DA (n=63) and CG (n=66) given a blank notebook<br><br>The study package contained the standard RAVES participant information and consent form with either the DA (IG) or a blank notebook (CG), which was the same size and shape as the DECISION AID so all packages had an identical weight and shape to maintain allocation concealment<br><br>92 participants (71%) were recruited by urologists and 37 (29%) by radiation oncologists<br><br>Recruitment rate 72%<br><br>Median age 63 years<br>Age range 42–74 years	Decisional conflict was significantly lower in the IG (DA) compared with the CG on average over the six-month study period ($p=0.048$); this mainly appeared to be due to lower IG scores in the sub-scales on feeling informed ($p=0.024$) and feeling supported ($p=0.025$) in their decision at one month<br><br>Knowledge on the RCT was significantly higher at 6 months ($p=0.033$) in the IG; 20.6% of the IG (13 out of 63) and 9% of the CG (6 out of 66) entered the RCT<br><br>Findings suggest that a decision aid may provide a way to supplement and standardise the quality and quantity of information provided to potential RCT participants, particularly those requiring multidisciplinary collaboration for recruitment<br><br>The proportion of men recruited to RAVES was more than double that in the DA arm (20.6%) than in the control arm (9%); this difference was not statistically significant

Reference	Study objective	Intervention	Study participants	Results of interest
		It also contained general information about RCTs, and the practicalities of RCT participation; value clarification exercises helped participants weigh participation in RAVES against receiving standard treatment		
**Tattersall et al. (2017)**<br>Parallel multicentre randomised trial of a clinical trial question prompt list in patients considering participation in phase 3 cancer treatment trials	To evaluate the effect of a clinical trial question prompt list in patients considering enrolment in cancer treatment trials	Pilot testing of a targeted QPL for patients with cancer considering clinical trial participation<br><br>Consenting patients were randomised to receive the clinical trial QPL (IG) or not (CG) before further discussion with their oncologist and/or trial nurse about the treatment trial<br><br>Questionnaires were completed at baseline and within three weeks of deciding on participation	88 patients with cancer attending three cancer centres in Australia, who were considering enrolment in phase 3 treatment trials<br><br>Intervention group (n=45)<br>Control group (n=43)<br><br>Detailed demographic and disease information, including the clinical treatment trial intervention included<br><br>Age range 22–85 years	88 out of 130 patients screened were enrolled (43 males), and 45 received the clinical trial QPL<br><br>49% of trials were chemotherapy interventions for patients with advanced disease; 35% were surgical adjuvant and 16% radiation adjuvant<br><br>70 patients completed all relevant questionnaires. 28 out of 43 patients in the CG compared with 39 out of 45 patients receiving the clinical trial QPL completed the QuIC ($p=0.0124$).<br><br>There were no significant differences in the QuIC scores between the randomised groups (QuIC part A $p=0.08$ and QuIC part B $p=0.92$).<br><br>There were no differences in patient satisfaction with decisions or in anxiety levels between the randomised groups
**Kamen et al. (2018)**<br>Multimedia psychoeducation for cancer patients eligible	To compare the effect of the MP intervention versus a PE intervention on:	An MP designed to improve patient attitudes toward clinical trials as well as provide education about clinical trials	418 patients with various types of cancer were recruited (age 26–89 years)	There were no significant differences between the MP and PE arms on the Preparation for Decision Making Scale after the intervention

Reference	Study objective	Intervention	Study participants	Results of interest
for clinical trials: a randomized clinical trial	1) decision support factors<br><br>2) attitudes toward clinical trials<br>3) willingness to participate in clinical trials	Assessments at baseline (before intervention), post-intervention, and at two-month follow-up visit<br><br>Patients randomised to the PE intervention met with a study coordinator who provided them with the National Cancer Institute booklet: Taking Part in Cancer Treatment Studies<br><br>Patients randomised to the MP intervention met with a coordinator who provided them with a DVD and booklet, both called Clinical Trials: Are They Right for You?	All diagnosed with cancer, eligible for a specific phase 2 or 3 therapeutic clinical trial (excluded if earlier in trial, excluded if only eligible for phase 1)<br><br>Most common cancer types: breast (29.2%)<br><br>Average standard deviation time since cancer diagnosis: 10.6–28.5 months<br><br>Women n=255 (61%)<br>Caucasian 89%<br><br>Patients were randomly assigned 1:1 by computergenerated random blocks of 4 to 1 of 2 intervention conditions:<br><br>1) a PE condition that included an informational booklet<br><br>2) an MP condition that included a DVD and a companion booklet	Patients randomised to the MP arm were significantly more likely to enroll in a clinical trial than those randomised to the PE arm<br><br>The difference between arms was not significant when comparing those who enrolled with those who did not enroll and who were undecided

**Polite et al. (2019)**<br><br>Investigation of a multimedia, computer-based approach to improve knowledge, attitudes, self-efficacy, and receptivity to cancer clinical trials among newly diagnosed patients with diverse health literacy skills	Test the effectiveness of an interactive health communication tool<br><br>Primary objective:<br><br>To improve patient receptivity, willingness, knowledge, self-efficacy and positive attitudes regarding therapeutic cancer clinical<br><br>Secondary objectives:<br>1) to evaluate the feasibility and acceptability of multimedia technology for PRO assessment and patient education in an oncology clinic<br><br>2) to evaluate the impact of the health communication tool on clinical trial discussion and enrollment	Patients viewed an interactive teaching video on clinical trials that was adapted from the National Institutes of Health: Taking Part in Cancer Treatment Research Studies<br><br>A letter was sent to the patients two weeks prior to their first visit with information on the intervention study<br><br>One week prior to the first visit a study coordinator contacted the patient with further information<br><br>Patients who were interested were asked to arrive 60–90 minutes before their appointment to consent to the intervention study and receive the intervention<br><br>Patients completed a pre-intervention survey<br><br>Patients used the talking touchscreen to complete a post-intervention survey	Patients (n=120) newly diagnosed with lung, gastric and pancreatic cancer were identified prior to their first oncologic visit at University of Chicago outpatient oncology clinics<br><br>Recruitment over 3 years (2014–2017)<br><br>Screened 59% of these patients (n=739), with informed consent obtained for 27%<br><br>Able to accrue 16% of those patients identified as eligible<br><br>Mean age 63 years<br><br>Diverse population:<br>80% non-Hispanic white<br>33% female<br>69% >high school education<br>8% reported an income <USD20,000<br><br>Approximately 33% scored within the low health literacy range	Passive decision making was found to be associated with less willingness to take part in a clinical trial compared with active decision making (p=0.021)<br><br>Patients with high health literacy demonstrated an increased willingness to take part in a clinical trial if one was offered to them (p=0.049); older patients demonstrated a decreased willingness (p=0.017)<br><br>The results suggest that multimedia technology has a role to play as an element of informed decision making for patients in that it did improve knowledge, self-efficacy and positive attitudes regarding clinical trials but likely needs to be coupled with more robust, multitargeted interventions to improve willingness and enrollment in therapeutic cancer clinical trials<br><br>Results fell into three categories:<br>1) active, in which the patient makes the decision alone or after considering the clinician's opinion (n=46, 46%)<br>2) shared, in which the patient makes the decision together with the clinician (n=40, 40%)<br>3) passive, in which the clinician makes the decision after considering the patient's opinion or alone (n=15, 15%)<br><br>63% of patients had discussed clinical trials with their physician after this study; of those patients who reported a discussion, about 38% were offered a clinical trial, and of those 48% enrolled in the trial<br><br>Non-Hispanic white patients were found to be twice as likely to discuss clinical trials with the physician at the time of their first oncology appointment, after their participation in the current study

Reference	Study objective	Intervention	Study participants	Results of interest
				(odds ratio 2.36; 95% confidence interval 0.95–5.86)<br><br>Patients who were married or had a partner were found to be three times as likely to discuss clinical trials with the physician compared with those having no partner (odds ratio 3.22; 95% confidence interval 1.25–8.30)<br><br>Despite an increase in willingness to take part in a clinical trial among those with high health literacy, there was no evidence that these patients were more likely to have discussed clinical trials with their physician

Abbreviations: ANZ: Australian/New Zealand group; ACT=Australian Cancer Trials; DA=Decision Aid; IG=Intervention group; HCP=Health care professionals; MP= Multimedia Psychoeducation; ns=No significant difference; PE=Print education; PRO=Patient-reported outcomes; QuIC=Quality of Informed Consent; RAVES=Radiotherapy – Adjuvant versus Early Salvage; RCT= randomized controlled trial, SDM=Shared decision making; SGA=Swiss/German/Austrian cohort; TT=Talking touchscreen; TTT=Teams Talking Trials; QPL–CT=Question Prompt List – Cancer Trial
